# MFG-E8, a clearance glycoprotein of apoptotic cells, as a new marker of disease severity in chronic obstructive pulmonary disease

**DOI:** 10.1590/1414-431X20154730

**Published:** 2015-09-08

**Authors:** S. Zhang, J.G. Xie, B.T. Su, J.L. Li, N. Hu, J. Chen, G.W. Luo, T.P. Cui

**Affiliations:** 1 Laboratory of Clinical Immunology, Wuhan No. 1 Hospital, Tongji Medical College, Huazhong University of Science and Technology, Wuhan, China; 2 Department of Respiratory and Critical Care Medicine, Wuhan No. 1 Hospital, Tongji Medical College, Huazhong University of Science and Technology, Wuhan, China; 3 Department of Respiratory and Critical Care Medicine, Tongji Hospital, Tongji Medical College, Huazhong University of Science and Technology, Wuhan, China

**Keywords:** Apoptosis, COPD, MFG-E8, Phagocytosis, Smoking

## Abstract

Milk fat globule epidermal growth factor 8 (MFG-E8) is an opsonin involved in the phagocytosis of apoptotic cells. In patients with chronic obstructive pulmonary disease (COPD), apoptotic cell clearance is defective. However, whether aberrant MFG-E8 expression is involved in this defect is unknown. In this study, we examined the expression of MFG-E8 in COPD patients. MFG-E8, interleukin (IL)-1β and transforming growth factor (TGF)-β levels were measured in the plasma of 96 COPD patients (93 males, 3 females; age range: 62.12±10.39) and 87 age-matched healthy controls (85 males, 2 females; age range: 64.81±10.11 years) using an enzyme-linked immunosorbent assay. Compared with controls, COPD patients had a significantly lower plasma MFG-E8 levels (P<0.01) and significantly higher plasma TGF-β levels (P=0.002), whereas there was no difference in plasma IL-1β levels between the two groups. Moreover, plasma MFG-E8 levels decreased progressively between Global Initiative for Chronic Obstructive Lung Disease (GOLD) I and GOLD IV stage COPD. Multiple regression analysis showed that the forced expiratory volume in 1 s (FEV_1_ % predicted) and smoking habit were powerful predictors of MFG-E8 in COPD (P<0.01 and P=0.026, respectively). MFG-E8 was positively associated with the FEV_1_ % predicted and negatively associated with smoking habit. The area under the receiver operating characteristic curve was 0.874 (95% confidence interval: 0.798-0.95; P<0.01). Our findings demonstrated the utility of MFG-E8 as a marker of disease severity in COPD and that cigarette smoke impaired MFG-E8 expression in these patients.

## Introduction

Chronic obstructive pulmonary disease (COPD) is a primary cause of chronic morbidity and mortality and has become one of the most serious diseases threatening human health throughout the world (1). Despite its prevalence, the precise pathogenesis of COPD is poorly understood. However, many studies have identified the pulmonary inflammatory response to cigarette smoke as the critical mechanism in COPD development. In addition, recent investigations have shown an association between COPD and both increased apoptosis and the reduced clearance of apoptotic cells (2).

Milk fat globule epidermal growth factor 8 (MFG-E8), a protein expressed mainly by activated macrophages and immature dendritic cells, is required for the phagocytosis of apoptotic cells as it links externalized phosphatidylserine present on the surface of apoptotic cells with integrins expressed on phagocytes (3). MFG-E8 thus plays a critical role in maintaining homeostasis by mediating the removal of apoptotic cells. Mice deficient in MFG-E8 develop a severe autoimmune and inflammatory disease resembling human systemic lupus erythematosus (SLE) (4). The aberrant expression of MFG-E8 has been demonstrated in several diseases, including SLE, atherosclerosis, and Alzheimer's disease (5-7). A common feature of these diseases is the defective phagocytic clearance of apoptotic cells. A deficiency in the ability of macrophages from patients with COPD to phagocytose apoptotic cells has also been reported (8). However, neither the relationship between MFG-E8 and defective efferocytosis in COPD nor the expression and function of MFG-E8 in COPD has been elucidated.

The aims of this study were to investigate the levels of plasma MFG-E8 in patients with COPD and to analyze the potential association between MFG-E8, disease severity, and smoking history.

## Material and Methods

### Ethics statements

This study was approved by the Ethics Committee of Wuhan No. 1 Hospital and Tongji Hospital, Tongji Medical College, Huazhong University of Science and Technology, Wuhan, China. All participants signed written informed consent, and the research complied with the Declaration of Helsinki and its amendments.

### Subjects

The 96 COPD patients and 87 healthy controls were recruited from the Wuhan No. 1 Hospital and Tongji Hospital, between March 2010 and March 2011. COPD was diagnosed according to the Global Initiative for Chronic Obstructive Lung Disease (GOLD) guidelines (1) and classified according to the GOLD criteria as: GOLD I (mild): FEV_1_(forced expiratory volume in 1 s)/FVC (forced vital capacity) <70%, FEV_1_ ≥80% predicted; GOLD II (moderate): FEV_1_/FVC <70%, 50%≤ FEV_1_<80% predicted, GOLD III (severe): FEV_1_/FVC <70%, 30%≤ FEV_1_ <50% predicted; GOLD IV (very severe): FEV_1_/FVC <70%, FEV_1_ <30% predicted. The 87 age-matched healthy controls without a smoking history (never smokers) were recruited from the Department of Medical Examination. Participants with a history of bronchial asthma, bronchiectasis, coronary heart disease, hyperlipidemia, cancer, autoimmune disease, serious brain, kidney or vascular disease, and inflammatory diseases were excluded.

The demographic and clinical characteristics of the patients and controls were obtained, including medical history and smoking habit (pack-years). Pulmonary function parameters (FEV_1_, FVC, and FEV_1_/FVC) were measured using a clinical spirometer. Each participant was required to perform the test three times and the mean measurement was determined.

### Sample collection and analysis

Blood was drawn from the peripheral vein into a vacutainer tube containing potassium-ethylenediaminetetraacetic acid as the anti-coagulant for plasma collection. The sample was then centrifuged for 10 min at 2000 g. Plasma was immediately removed, aliquoted, and stored at -80°C until analysis. Plasma levels of MFG-E8, IL-1β and TGF-β were measured with commercial ELISA kits (MFG-E8, Cusabio, China, Cat. CSB-E12637h; IL-1β, R&D, USA, Cat. DY201; TGF-β, R&D, USA, Cat. DY240). All samples were measured in triplicate.

### Statistical analysis

All statistical analyses were carried out using the SPSS software, version 18.0. The data are reported as the mean±SE. Two independent groups with normal and non-normal distributions were analyzed using Student's *t*-test and the Mann-Whitney U*-*test, respectively. The χ^2^ test was used to compare proportions in different groups. One-way ANOVA and the Kruskal-Wallis H test were used to compare more than two groups depending on their distribution status. Multiple linear regression was used to evaluate the relationship between the levels of plasma MFG-E8, different pulmonary function indicators, and confounding factors such as age, body mass index (BMI), and smoking history. A stepwise method was applied to derive the final model. The inclusion and exclusion criteria of independent variables were P≤0.05 and P≥0.10, respectively. The receiver operating characteristic (ROC) curve for the various MFG-E8 cut-off values, as a predictor of COPD, was also obtained. Youden's index was calculated (YI=sensitivity+specificity-1) to determine the cut-off value with the maximum sensitivity and specificity.

## Results

### Clinical characteristics of the study participants

Among the 96 COPD patients, 5 patients had GOLD I, 40 had GOLD II, 34 had GOLD III, and 17 had GOLD IV disease. The baseline characteristics of these patients and the 87 healthy controls are reported in Table 1. The two groups did not differ significantly with respect to age, gender, and BMI. However, the pulmonary function tests revealed a significantly lower FEV_1_, FVC, FEV_1_/FVC, and FEV_1_ % predicted in COPD patients than in the control group (Table 1).

**Table:**
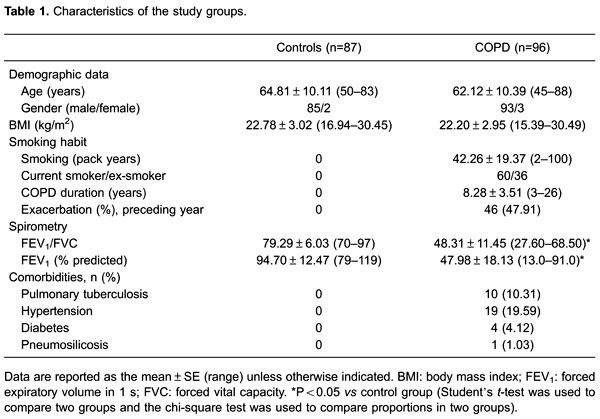

### Plasma levels of MFG-E8 in COPD patients vs healthy controls

**Figure 1:**
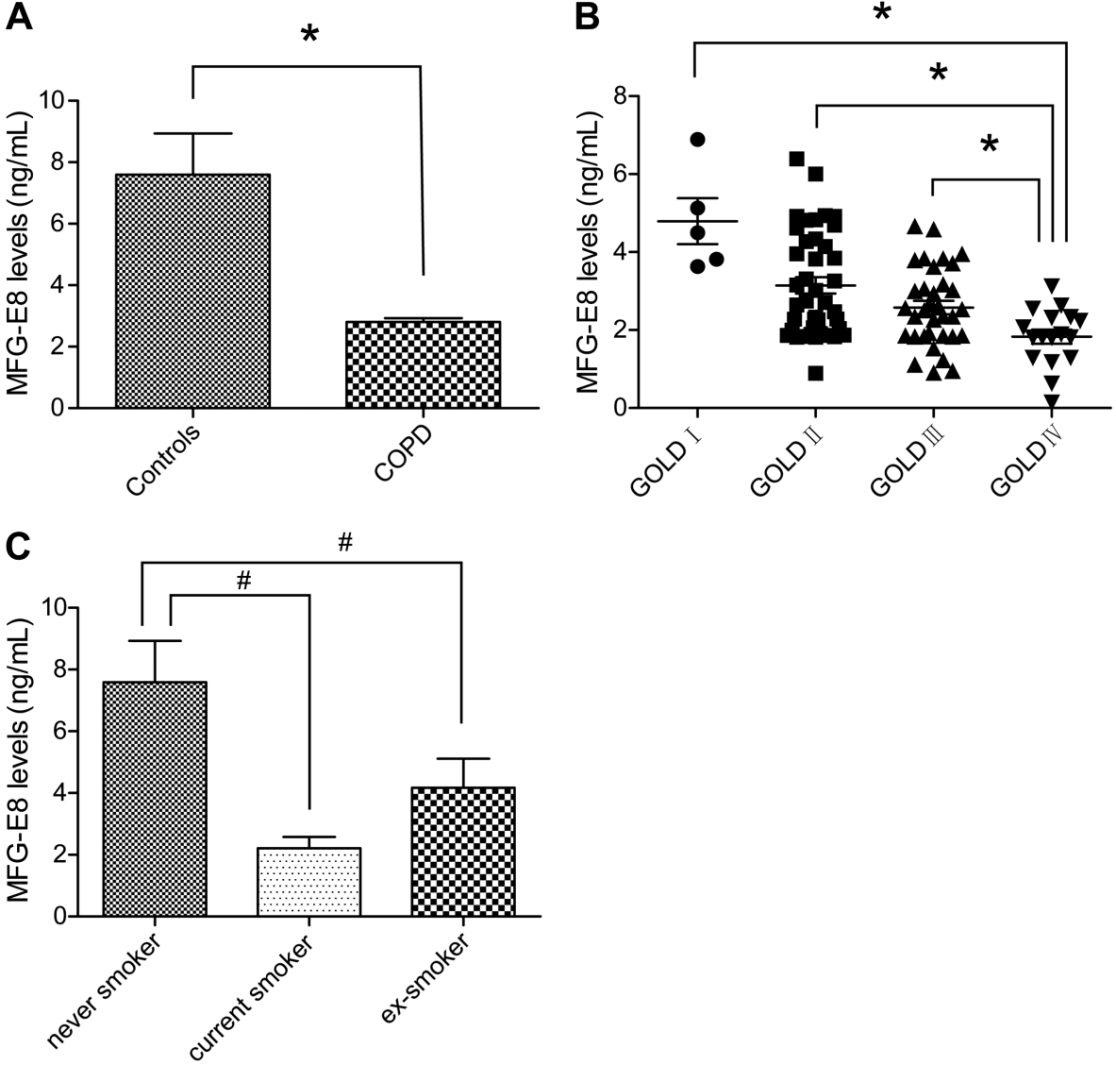
*A*, Plasma milk fat globule epidermal growth factor 8 (MFG-E8) levels in healthy controls and patients with chronic obstructive pulmonary disease (COPD). *B*, Plasma MFG-E8 levels in COPD patients differing in the severity of their disease. Global Initiative For Chronic Obstructive Lung Disease (GOLD) criteria: GOLD I (mild); GOLD II (moderate); GOLD III (severe); GOLD IV (very severe). *C*, Plasma MFG-E8 levels among never, current, and ex-smokers. *A*: *P<0.05 *vs*control; *B*: *P<0.05 *vs* GOLD IV; *C*: #P<0.05 *vs* never smoker (Mann-Whitney U*-*test was used to compare two groups and Kruskal-Wallis H test was used to compare more than two groups).

Plasma levels MFG-E8 were much lower in patients with COPD than in the control group (2.76±0.13 *vs* 7.60±1.34 ng/mL; P<0.01; Figure 1A). Moreover, there were clear differences in the plasma MFG-E8 levels of COPD patients with different disease severity as evaluated using the GOLD criteria. Specifically, the greater the disease severity, the lower the plasma MFG-E8 level (Table 2). As shown in Figure 1, compared with the GOLD IV group, plasma MFG-E8 levels were markedly higher in the GOLD I, II, and III groups (P values of 0.025, <0.01, and 0.027, respectively; Table 2). However, the differences in plasma MFG-E8 levels between the GOLD I and GOLD II groups and between the GOLD II and GOLD III groups were not significant (both P>0.05; Figure 1B). By contrast, MFG-E8 levels in plasma were significantly lower in patients who were current and ex-smokers than in never smokers (healthy controls) (2.21±0.37, 4.15±0.93, and 7.59±1.34 ng/mL, respectively; P<0.01). The difference between current and ex-smokers was not significant (P>0.05; Figure 1C).

**Table:**
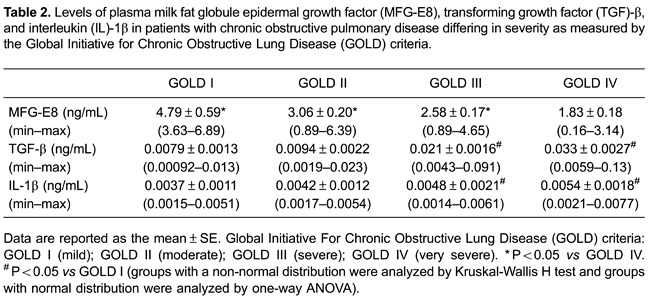

### Variables associated with plasma MFG-E8

**Figure 2:**
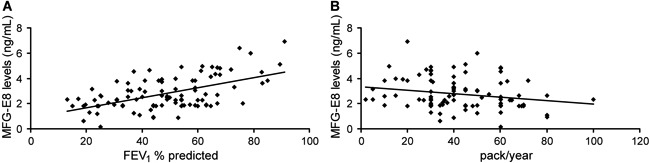
Correlation analysis between milk fat globule epidermal growth factor 8 (MFG-E8), forced expiratory volume in 1 s (FEV_1_) % predicted, and smoking habit.

The associations between plasma MFG-E8 levels, pulmonary function indicators, and confounding factors such as BMI and smoking history were investigated using a stepwise multiple regression analysis. The results showed that only two variables, FEV_1_ % predicted and the amount of smoking, fit the regression equation (P<0.01 and P=0.026, respectively; Table 3). Moreover, plasma MFG-E8 levels were positively associated with the FEV_1_ % predicted (Figure 2A) and negatively associated with smoking habit (Figure 2B). BMI and the pulmonary function indicators FEV_1_/FVC, FVC, and FEV_1_, as potentially confounding factors, had no effects on the plasma levels of MFG-E8 (P≥0.1).

**Table:**
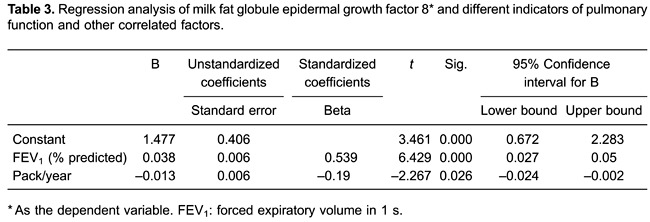

### Plasma IL-1β and TGF-β levels in COPD patients *vs* healthy controls

**Figure 3:**
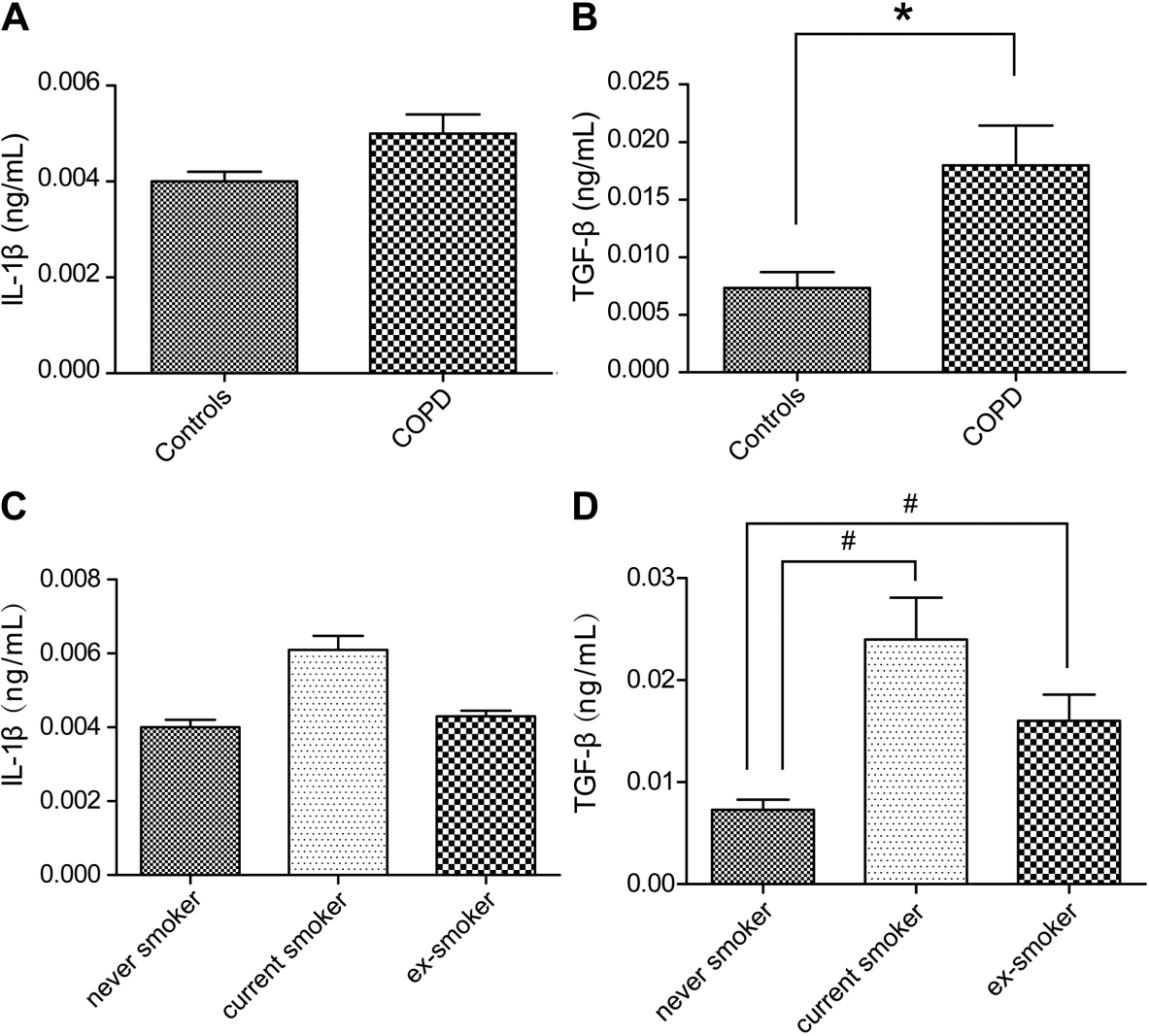
*A*, Plasma interleukin (IL)-1β levels in healthy controls and patients with chronic obstructive pulmonary disease (COPD). *B*, Plasma transforming growth factor (TGF)-β levels in healthy controls and COPD patients. *C*, Plasma IL-1β levels among never, current, and ex-smokers. *D*, Plasma transforming growth factor (TGF)-β levels among never, current, and ex-smokers. *P<0.05 *vs* controls; ^#^P<0.05 *vs* never smoker (Student's *t*-test was used to compare two groups and one-way ANOVA was used to compare three groups).

MFG-E8 is thought to attenuate inflammation by clearing apoptotic cells, such that a reduction in MFG-E8 production should be associated with an increase in the pro-inflammatory response. To confirm this hypothesis, we measured the plasma levels of IL-1β and TGF-β, as markers of inflammation, in patients with COPD. The results showed that the plasma levels of the pro-inflammatory cytokine IL-1β did not significantly differ between COPD patients and healthy controls (0.005±0.004 *vs*0.004±0.002 ng/mL; P>0.05) nor among COPD patients with different disease severity according to the GOLD criteria (P>0.05; Table 2). Likewise, there was no significant difference among never smokers, current smokers, and ex-smokers (P>0.05; Figure 3C). By contrast, plasma TGF-β levels were significantly higher in COPD patients than in healthy controls (0.018±0.0035 *vs* 0.0073±0.0014 ng/mL, P=0.002; Figure 3B), in patients with GOLD III and IV *vs* GOLD I and II disease (P<0.01; Table 2), and in current and ex-smokers than in never smokers (P<0.01 and P=0.012, respectively; Figure 3D).

### ROC curve analysis

**Figure 4:**
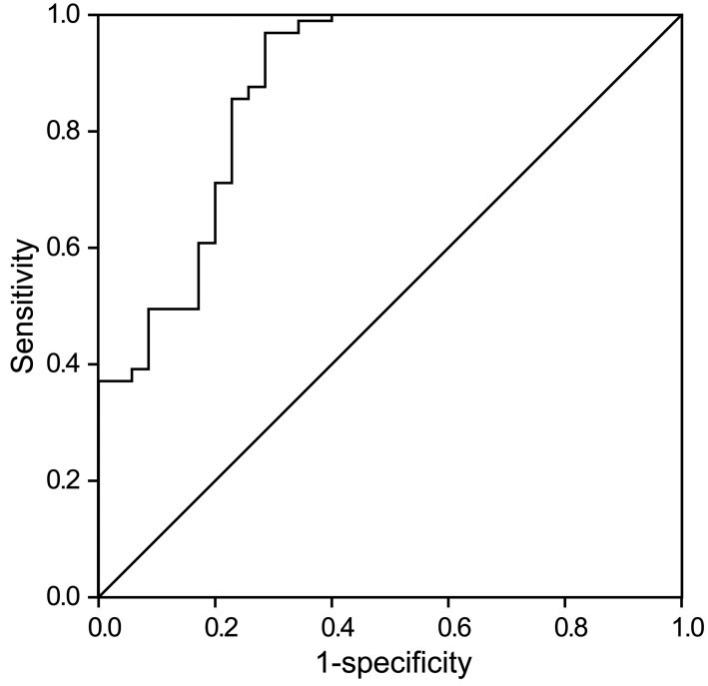
Receiver operating characteristic (ROC) curve of milk fat globule epidermal growth factor 8 (MFG-E8) in diagnosing chronic obstructive pulmonary disease (COPD). The area under the ROC curve was 0.874 (95% confidence interval: 0.798-0.95; P<0.01). The MFG-E8 cut-off value in predicting COPD was 5.27 ng/mL (sensitivity 96.9%, specificity 71.4%).

In a ROC curve analysis, the area under the curve was 0.874 and the 95% confidence interval was 0.798-0.95 (P<0.01). The MFG-E8 cut-off value in predicting COPD was 5.27 ng/mL (sensitivity 96.9%, specificity 71.4%; Figure 4).

## Discussion

Apoptosis, in which damaged or unwanted cells are removed, is critical to the maintenance of homeostasis. The recognition and removal of apoptotic cells is mediated by a variety of molecules, including receptors on the phagocyte surface and soluble bridging proteins such as MFG-E8, thrombospondin-1, and protein S. Both an increase in the number of apoptotic cells and defective efferocytosis are involved in the pathogenesis of COPD (8-10). However, their exact contributions are poorly understood. Hodge et al. (8) reported that a decrease in the expression of phagocyte surface receptors, such as CD31, CD91, and CD44, is responsible for defective efferocytosis in COPD (11). In the present work, we asked whether the altered expression of the soluble bridging protein MFG-E8 is associated with defective efferocytosis in COPD.

Our result showed that plasma MFG-E8 levels were distinctly lower in patients with COPD than in healthy controls. Moreover, the concentration of MFG-E8 in COPD patients decreased progressively from GOLD I to GOLD IV, suggesting a negative correlation between MFG-E8 and the severity of COPD. This relationship was confirmed in a multiple regression analysis, which showed that plasma MFG-E8 levels correlated positively with FEV_1_ % predicted. According to these data MFG-E8 may be a useful marker to evaluate disease severity in COPD. Moreover, the ROC curve analysis showed that the cut-off value for MFG-E8 was a good diagnostic indicator of COPD.

Consistent with our findings, the reduced expression of MFG-E8 in other diseases characterized by defective efferocytosis, such as atherosclerosis, has been reported. In the study of Dai et al. (6), plasma MFG-E8 levels were lower in patients with coronary atherosclerotic heart disease and MFG-E8 expression was reduced in atherosclerotic plaques. In another study, MFG-E8 was shown to facilitate the phagocytic clearance of apoptotic cells, and in atherosclerotic mice deficient in MFG-E8 there was both a substantial accumulation of apoptotic cells and an increase in atherosclerotic lesion size (12). MFG-E8 has been implicated in Alzheimer's disease as well, based on a significant reduction in its expression in the brain tissue of these patients (7). The same authors found that MFG-E8 was involved in the phagocytosis of amyloid β-peptide. Their results indicate that alterations in MFG-E8 production contribute to the initiation of Alzheimer's disease (7). Similarly, in COPD patients, the reduced expression of MFG-E8 may reflect its increased consumption during the removal of the large numbers of apoptotic cells present in the lungs and in bronchial tissue.

Because cigarette smoking is the major cause of COPD, a multiple regression analysis was performed to determine whether this relationship involved the decreased expression of MFG-E8. The results showed a negative correlation between MFG-E8 and the amount of smoking. Cigarette smoke impairs the clearance of apoptotic cells by phagocytes (13,14) and thus may inhibit the expression of macrophage surface molecules that recognize apoptotic cells (11). Accordingly, cigarette smoke may impair macrophage expression of the apoptosis bridging protein MFG-E8 in patients with COPD.

Inflammation is an important mechanism in the development of COPD and MFG-E8 could attenuate inflammation indirectly by removing apoptotic cells. There is also an increasing body of evidence supporting a direct anti-inflammatory role of MFG-E8 in inflammatory diseases such as acute lung injury, sepsis, and colitis (15-17). MFG-E8 reduces the expression of pro-inflammatory cytokines, including IL-1, IL-6, and TNF-α. To determine whether the low-level expression of MFG-E8 in COPD is coupled to an increased inflammatory response, we measured the levels of plasma IL-1β and TGF-β in patients with COPD. While the levels of the anti-inflammatory cytokine TGF-β were markedly elevated, the difference in the levels of the pro-inflammatory cytokine IL-1β did not significantly differ from those of healthy controls. This observation is in agreement with a recent finding of increases in TGF-β and apoptotic T lymphocytes in the peripheral blood of COPD patients (18). The authors suggested that the increase in TGF-β was involved in inducing apoptosis by T cells and alveolar wall cells, via binding to the TGF-β receptor 1, thereby leading to the development of emphysema.

Our study had two major limitations. First, it may have been useful to measure the expression of MFG-E8 in lung tissue or in bronchoalveolar lavage fluid. Second, the effect of statins, corticosteroids, and other drugs on the expression of MFG-E8 was not determined. Nonetheless, our study is the first to report a decrease in plasma MFG-E8 levels in patients with COPD and to demonstrate a negative association between MFG-E8 and the severity of COPD. Thus, measuring the changes in plasma MFG-E8 may be a promising method for evaluating the severity of COPD.
